# The Surgical Practice at the Hôtel Dieu

**Published:** 1842-11-05

**Authors:** 


### ANALECTA.

*The Surgical Practice at the Hôtel Dieu.*—In one of the late numbers of the French Medical Gazette there is a statistical report for 1841, of the surgical practice of Professor Roux at the Hôtel Dieu in Paris—the largest Hospital, it is well known, in that metropolis.

The total number of patients admitted under his care, during the 12 months, was 1441—of whom 1303 were males and 138 were females.

The operations performed were as follows :—

| | Cases. | Deaths. |
|---|---|---|
| Amputation of the thigh, | 8 | 5* |
| Amputation of the leg, | 5 | 3 |
| Amputation supra-malleolar, | 1 | 0 |
| Amputation of the fore-arm, | 2 | 1 |
| Amputation of the metacarpal bones, | 4 | 2 |
| Amputation of the fingers, | 7 | 0 |
| Resection of the elbow, | 3 | 1 |
| Resection of a rib, | 1 | 0 |
| Extraction of Sequestræ, | 2 | 0 |
| Trepan, | 2 | 2 |
| Cancer of the lower lip, | 3 | 0 |
| Epulis, | 1 | 0 |
| Cancer of the cheek, | 1 | 0 |
| Section of the genio-glossi for stammering, | 1 | 1 |
| Cancer of the tongue, | 2 | 0 |
| Cancer of the velum palati, | 1 | 0 |
| Staphyloraphy, | 3 | 0 |
| Excision of the tonsils, | 4 | 0 |
| Excision of an osseous cyst on the upper jaw, | 1 | 0 |
| Hernia, crural, | 6 | 5 |
| Hernia, inguinal, | 3 | |
| Fissure of the anus, | 18 | 0 |
| Fistula, recto-vaginal, | 1 | 0 |
| Anus, imperforation of, *Littre's* operation, | 1 | 1 |
| Anus, at umbilicus, | 1 | 1 |
| Amputation of the penis, | 1 | 0 |
| Amputation of the testicle, | 3 | 0 |
| Lithotomy, | 4 | 3 |
| Lithotrity, | 2 | 1 |
| Excision of the mamma, | 6 | 0 |
| Strabismus, | 6 | 0 |
| Cataract, | 41 | 0 |
| Artificial pupil | 1 | 0 |
| Varicocele, | 5 | 2 |

With several others of minor importance.

The total number of deaths that occurred after operations was 34. If we add to this 56, the number of cases which proved fatal spontaneously, either from the severity of the accident sustained, or the gravity of the existing disease, we find the entire mortality to be ninety-one during the twelve months.

By far the most frequent cause of death after the operations, was purulent absorption, and the consequent contamination of the whole system, the induction of hectic fever, &c.

If we may judge from the result of one year's experience, it would seem that cold weather is decidedly unfavourable to the success of great opera-

* In one case both thighs were amputated.

tions: nearly two-thirds of the deaths occurred during the months of January and February.

Erysipelas, it deserves to be noticed, was by no means very prevalent during this year; the absence of this most pernicious visitant may perhaps account for the comparative smallness of the mortality during the warm months.

There is very little interest in the separate details of this report; it is meagre and unsatisfactory. As we have heard so much for the last year or two of the *brilliant results* of myotomy, applied to various diseases, we select the following notice of M. Roux's practice in a case or two of stammering.

The Reporters state with not a little justice that, even before the results of surgical interference in the treatment of this complaint were at all ascertained, a variety of operations had been proposed and adopted by different men. Unfortunately for them all, they seem to have now fallen into complete oblivion.

M. Roux, as a matter of course, was called upon to test the real merits of the cutting method of treating impediments of speech. In one case he divided merely the frænum of the tongue—in an old man, 60 years of age—which was perfectly free in its movements. This man certainly stammered most shockingly, all the muscles of his face being often thrown into convulsive spasms, when he made an effort to speak. No sooner was the frænum divided, than "avec beaucoup de netteté il se confond en remercimens;" and he naturally expressed his astonishment that he should have been allowed to remain all his lifetime without relief. The cure seemed to every one at the time to be "brilliant and complete;" when, behold, he began to stammer as bad as ever, and in this state he remained during all the time that he was in the hospital.

In another case, M. Roux divided the *genio-glossi* muscles at their insertion into the lower maxilla; here too the stammering ceased at the time, but speedily returned; and when he left the hospital, he was no better than before.—*Medico-Chirurgical Review.* Oct., 1842.

